# Tailoring and characterization of bioactive graft material for alveolar bone preservation and regeneration in fresh extraction sockets of dog model

**DOI:** 10.1038/s41598-025-86408-x

**Published:** 2025-01-27

**Authors:** Ayat Hamdy, Manal Saad, Rania ElBackly, Samir Nouh, Himanshu Jain, Mona Marei

**Affiliations:** 1 Tissue Engineering Laboratories, Faculty of Dentistry, Alexandria University, Alexandria, Egypt; 2 Oral and Maxillofacial Surgery Specialist, Ministry of Health, Alexandria, Egypt; 3 Oral Biology Department, Ahram Canadian University, 6th of October City, Egypt; 4 Endodontics, Conservative Dentistry Department, Faculty of Dentistry, Alexandria University, 13 Champolion St., Azarita, Alexandria, Egypt; 5 Veterinary Surgery, Faculty of Veterinary Medicine, Alexandria University, Alexandria, Egypt; 6 Institute for Functional Materials and Devices (I-FMD), P.C. Rossin College of Engineering and Applied Science, Lehigh University, Bethlehem, PA USA; 7 Removable Prosthodontics Department, and Founder of the Tissue Engineering Laboratories, Faculty of Dentistry, Alexandria University, Alexandria, Egypt

**Keywords:** Bioactive glass, Dynamic tissue regeneration, Alveolar bone defect, Multi-porous scaffold, Degradable biomaterials, Osseointegration, Diseases, Medical research, Materials science, Prosthetic dentistry, Health care, Dentistry, Dental implants, Removable prosthodontics

## Abstract

The objective of this study was to tailor an osteoinductive scaffold for alveolar bone regeneration and around immediately placed implants in extraction sockets of dogs. Tailored amorphous multiporous bioactive glass (TAMP -BG) was prepared and characterized for bioactivity and response of human alveolar bone marrow mesenchymal stem cells (hABMSCs). Extraction sockets of twenty-two male mongrel dogs received TAMP-BG in the right side around implant in the distal socket of the mandibular fourth premolar (P4), while the adjacent empty mesial socket of the same tooth was filled with the same graft. Autologous bone chips were used in the left side. Animals were euthanized at 1, 2, 4, 8 &12 weeks for histological and SEM analyses. SEM/EDX, FTIR and XRD analyses displayed formation of a bioactive hydroxycarbonated apatite layer. TAMP-BG significantly increased proliferation and migration of hABMSCs. Histologically, at the 1^st^ week, advanced bone healing was shown in TAMP-BG group. At four weeks bone density was significantly higher forTAMP-BG (92.71% ± 1.71) versus control (62.92% ± 2.02) (P = 0.0001).At eight weeks, significant increase in width of buccal plate and height of lingual bony plate was observedfor TAMP-BG grafted implant.The socket orifice width significantly decreased for autologous bone from 1 to 12 weeks (P < 0.001), while it significantly increased for TAMP-BG (P = 0.03). We conclude that TAMP-BG can provide a preservative dynamic microenvironment following extraction up to three months which can be attributed to its unique physico-chemical characteristics.

**Supplementary Information:**

The online version contains supplementary material available at 10.1038/s41598-025-86408-x.

## Introduction

Data from human clinical trials have shown that clinical loss in width of the alveolar ridge following tooth extraction becomes more than the loss in height, assessed both clinically and radiographically^1,2^. Alveolar ridge preservation via socket grafting may reduce these physiological changes^3,4^. During the past 2 decades, numerous alveolar ridge preservation techniques and materials have been proposed. Various grafting materials were introduced including autologous bone^5^, allografts^6,7^, xenografts^8–10^ and alloplasts^11,12^***.*** However, a systematic review and meta-analysis in 2014^13^ highlighted that the grafting material may either impair or accelerate the normal wound healing response based on its characteristics particularly since the rate of ridge resorption is highest during the first 3 months following extraction. Furthermore, the review concluded that while changes in alveolar ridge dimensions were reduced, none of the grafting materials was able to prevent ridge resorption. Therefore, the authors emphasized the importance of developing and characterizing novel biomaterials capable of enhancing healing of extraction sockets and at the same time limit the negative effects of the remodeling process to provide more predictable outcomes following later implant placement. A recent review also demonstrated that while regenerative graft materials maybe beneficial; there is still a need for conclusive evidence from well-conducted clinical trials^14^.

Tissue engineering offers a platform for tailoring and designing biomaterials with diverse biological and physical characteristics that can modulate the process of bone regeneration based on a profound understanding of the specificity of the native tissue as a prerequisite^15–17^.

**Fig. 1:**
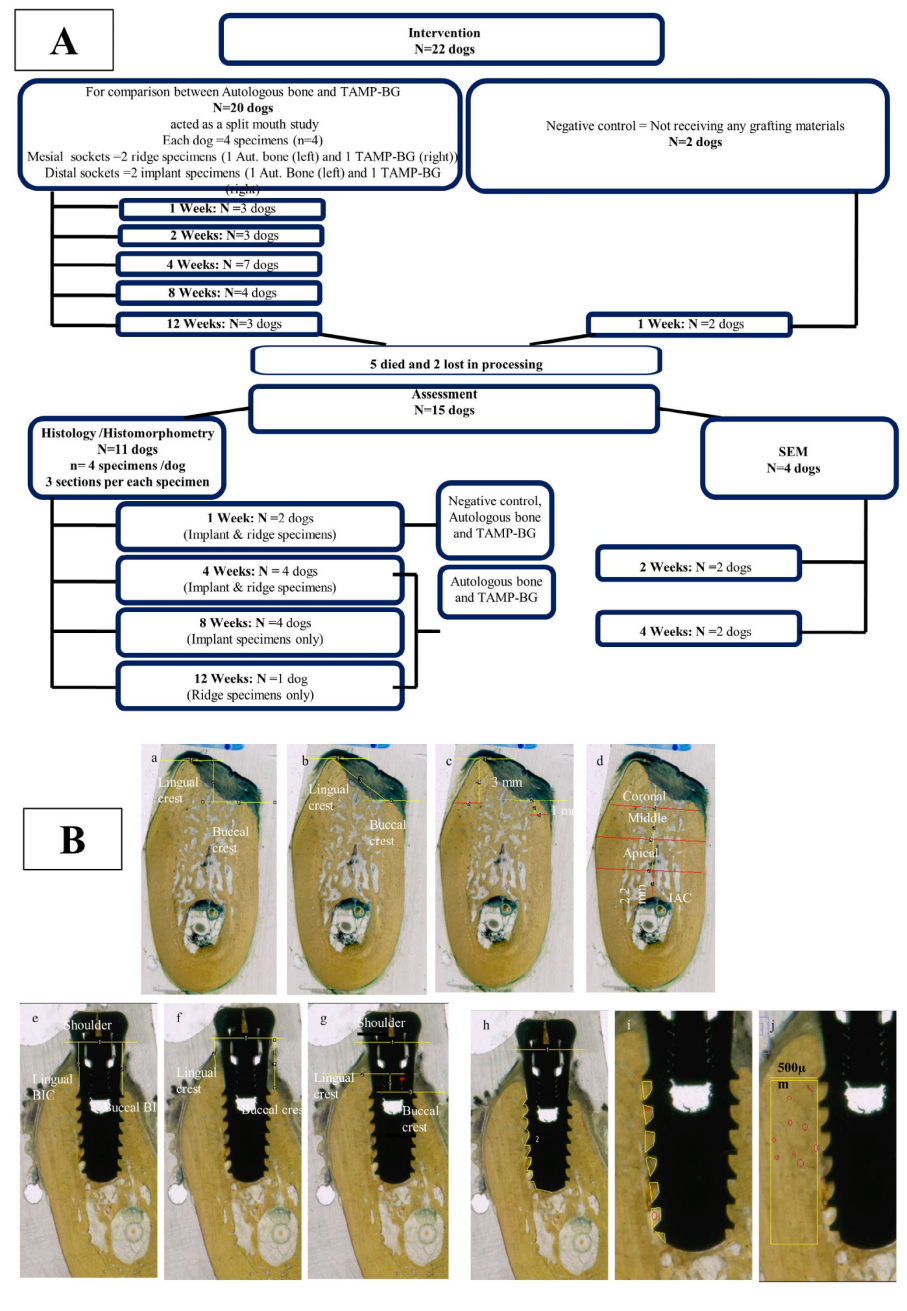
Panel (A) shows Flow chart of animal experiments; representing study groups and numbers of animals used in each interval and assessment. Panel (B) shows the scanned images of specimens sections representing histomorphometric analysis of alveolar ridge sockets and implant specimens; where a-d represent ridge specimens, while e-j represent implant specimens. (**a**) The measurements of difference between buccal and lingual heights (vertical yellow line). (**b**) The coronal contour (linear measurement at the orifice of the socket) from the highest buccal point to the highest lingual one (buccal & lingual crests). (**c**) The lingual and buccal plates widths at 1& 3 mm levels (red lines). (**d**) The total bucco-lingual area at coronal, middle and apical areas (red lines). (**e**) The measurements of distance from implant shoulder to 1st BIC. (**f**) The distance from the implant shoulder to the lingual and buccal bone crests. (**g**) The difference between buccal and lingual heights (red asterisk). (**h**) The bone implant contact (BIC) %. (**i**) The bone fill % within the implant threads. (**j**) The bone fill % outside the threads area for a distance of 500 µm lateral to implant threads (red circles are bone marrow areas).

Bioactive glasses were introduced by Hench in 1969^18^. They proved to undergo a particular biological reaction at the interface of bone, which stimulates cell proliferation, gene response and the formation of a bond between living tissues and the material. The soluble ions released by these materials during their degradation and their ability to strongly bond to bone is referred to bioactivity which is mediated through the formation of a surface layer of carbonated hydroxyapatite (HCA)^18,19^. The bimodal nano–macro porosity of bioactive glasses has shown desirable requirements of degradation rate suitable for bone tissue engineering^20–22^. In this context, the material’s characteristics must be critically examined. Scanning electron microscopy (SEM/EDX) examines a material’s surface topography, porosity and its distribution, as well as its elemental composition, all of which affect ion dissolution and bioactivity which in turn influence cellular and molecular interactions. Fourier transform infrared (FTIR) spectroscopy is a specific analysis of the functional groups that gives a material its unique characteristics. X-ray diffraction (XRD) analysis gives an indication about the crystal structure of the material; which both affects the material’s degradation that is followed by bioactive layer formation which is a key factor in the material’s bioactivity and interactions with the surrounding tissues^22,23^.The primary outcome of the current investigation was to tailor and characterize 3/D amorphous multi-scale porous bioactive glass scaffold “TAMP-BG” to be utilized as a bioactive graft material. The secondary outcome was to study the effect of this graft on alveolar bone preservation and new bone formation around immediately placed implants in a dog model compared to autologous bone graft (Fig. 1).

## Results

### Dissolution and bioactivity evaluation of TAMP-BG (Supplementary Figs. 1 & 2)

#### pH and (ionic concentration) ICP results

**Fig. 2:**
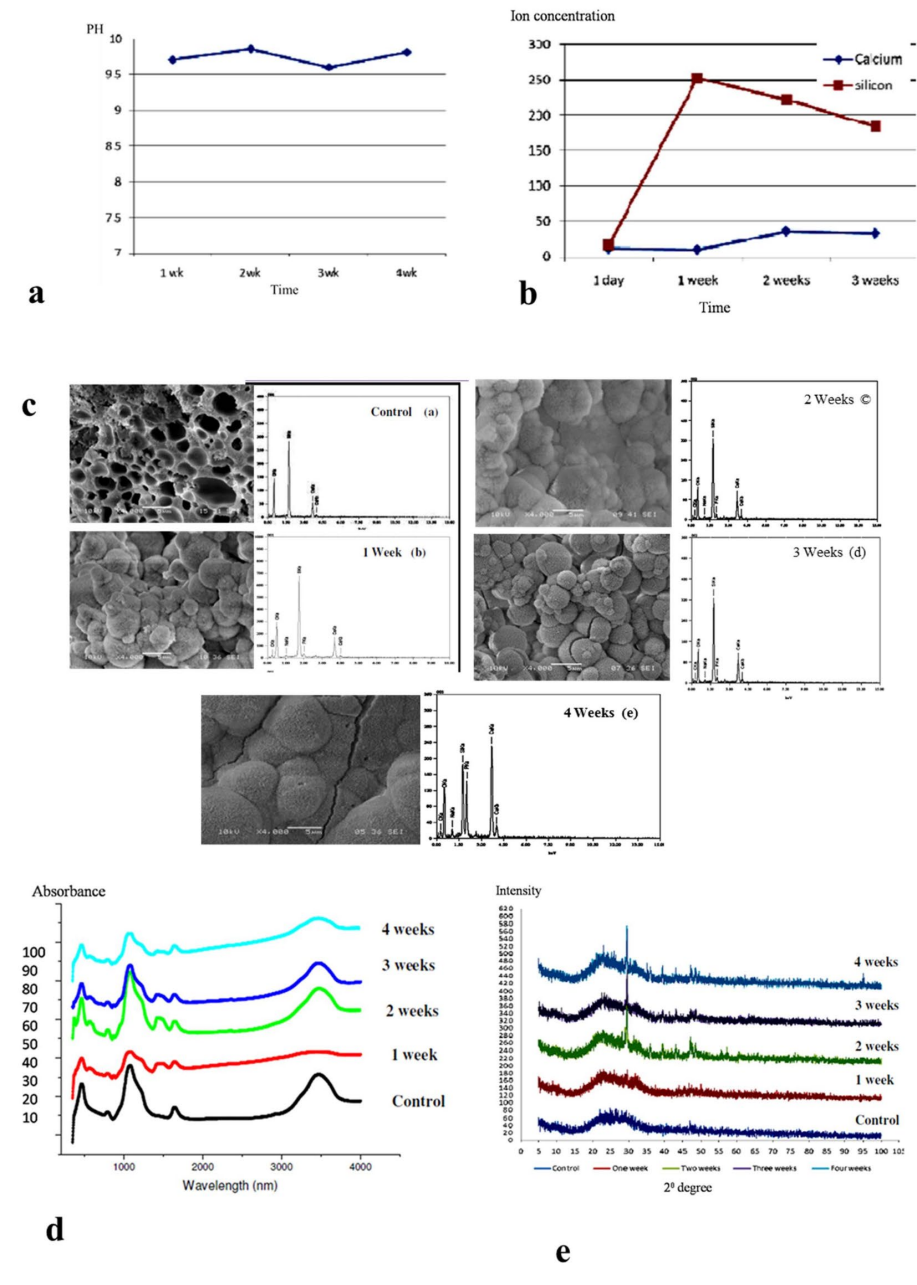
Dissolution and bioactivity evaluation of TAMP-BG. (**a**) The pH values measured after ions release have shown all samples with an alkaline pH more than 9.5, after being soaked in PBS for 1, 2 and 3 weeks. (**b**) Graphical representation of Si and Ca ions release in PBS up to 3 weeks (**c**) SEM/EDX micrographs/spectra showing the formation and development of the HCA layer on the TAMP-BG surface starting from one week until the 4th week. (**d**) FTIR spectra of the TAMP-BG scaffolds before and after soaking in PBS as a function of time, showing the development of HCA peaks. (**e**) XRD spectra of the scaffolds before and after soaking in PBS as a function of time showing the development of HCA peaks.

All samples recorded pH more than 9.5. Silicon release increased till the first week then decreased at the following weeks but was still higher than the first day. Calcium release remained the same in the first week, and then it increased significantly in the second week, finally, it remained stable in the third week (Fig. 2a & b).

#### SEM/EDX results

A gel-like layer formed on the sample surfaces. At 4 weeks, this layer became richer in Calcium (Ca) and Phosphorus (P) and less rich in Silicon (Si). Needle shaped crystals and cracks were seen on the surface (Fig. 2c). In vivo experiments under the skin in rabbits displayed more rapid biodegradation rate of TAMP-BG than in vitro degradation reflecting the influence of the in vivo dynamic environment on the scaffold (Supplementary Figs. 1 & 2).

#### FTIR analysis

The infrared spectra of the glass samples before and after immersion in PBS for 4 weeks are shown in (Fig. 2d). The peaks at 466, 794, and 1083 cm-1 correspond to Si–O-Si, whereas at 1083 and 1644 cm^-1^ correspond to (H–O). With increasing soaking time, new peaks appeared at 565 cm^-1^ corresponding to (P–O). While those at 874 and 1476 cm^-1^ could be assigned to (C–O).

#### X-Ray diffraction results

By 4 weeks of soaking, a gradually developed peak was observed at an angle ~ 29.8 and (104) plane indicating the formation of HCA layer (Fig. 2e).

### Evaluation of the effect of TAMP-BG extract on migration and proliferation of hABMSCs

#### Analysis of TAMP-BG extract

**Fig. 3:**
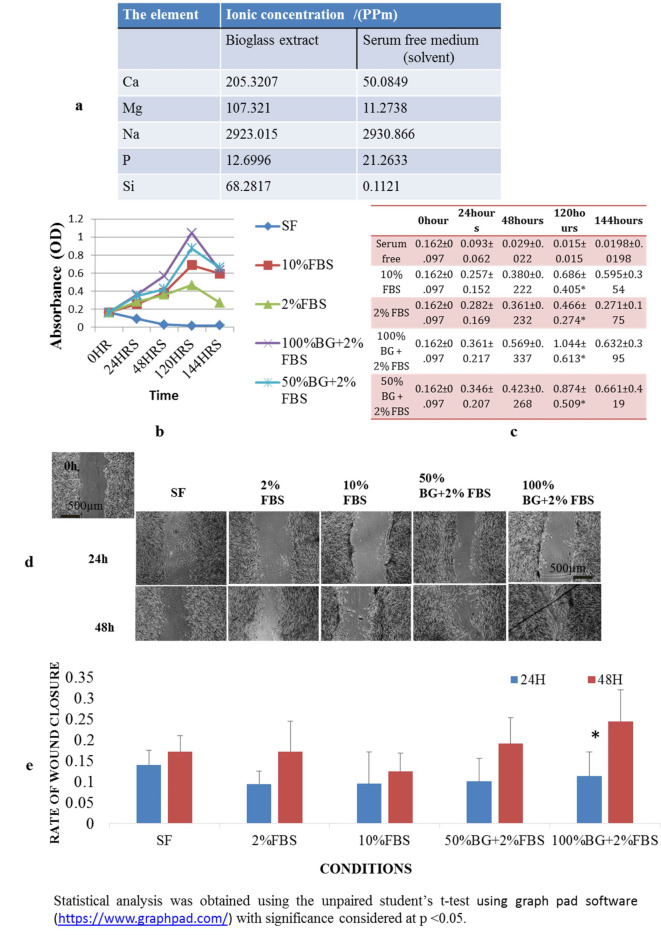
Evaluation of the effect of TAMP-BG extract on migration and proliferation of human alveolar bone marrow-derived mesenchymal stem cells. (**a**) Table representing ionic concentration (/PPm) in TAMP-BG extract, in comparison to the serum free medium. (**b**) Graphical representation of mean values of absorbance for each condition (SF, 10%FBS, 2%FBS, 100% BG + 2% FBS and 50% BG + 2% FBS) in the MTT assay at different time points (0, 24, 48, 120 and 144 h). (**c**) Table of mean values ± standard deviations [seen in (b). (**d**) Wound scratch assays with cell migration of hABMSCs in response to TAMP-BG extracts after 24 and 48 h. Scale bar = 500 μm .(**e**) Rate of wound closure of scratched hABMSCs in response to TAMP-BG extracts after 24 and 48 h. Statistically significant differences are demonstrated by the (*) on top of columns.

TAMP-BG extract showed an increase in the levels of Ca, Mg and Si ions and a decrease in the concentration of P while, the concentration of Na remained the same (Fig. 3a).

#### Cell proliferation using MTT

Cell proliferation was enhanced for all conditions as compared to serum free controls with a significant difference after 120 h. TAMP-BG extract regardless of concentration non-significantly enhanced proliferation with a higher rate compared to the other culture conditions (Fig. 3b&c).

#### Wound scratch migration results

Migration rate was increased in presence of TAMP-BG extract. At 48 h TAMP-BG with 100% concentration significantly showed the highest rate of wound closure compared to the other conditions of different culture media and durations (Fig. 3d&e).

### Histology and histomorphometric results

#### The ridge specimens (the empty mesial extraction socket of P4 filled with graft)

Results of histomorphometric measurements are documented in Table 1 and Supplementary Table 1.

**Table 1:** Comparison between the autologous bone and TAMP-BG groups of ridge specimens at different time points; regarding the difference between buccal and lingual heights, the coronal contour (linear measurement at the orifice of the socket), the buccal and lingual plates’ widths at 1, 3 & 5 mm levels and the overall buccolingual coronal, middle and apical areas.

			Autologous bone	TAMP-BG	T-test P value
			Mean ± SD		
L-B crestal height		1 week	-0.71 ± 0.26	0.12 ± 0.45	0.02*
		4 weeks	0.21 ± 0.03	0.12 ± 0.00	0.04*
		12 weeks	-0.80 ± 0.37	-2.31 ± 0.56	0.02*
		P value	0.003*	< 0.001*	
Coronal contour (orifice)		1 week	4.70 ± 0.15	3.97 ± 0.33	0.02*
		4 weeks	3.55 ± 0.26	4.38 ± 0.61	0.09
		12 weeks	3.19 ± 0.41	5.04 ± 0.05	0.002*
		P value	< 0.001*	0.03*	
Buccal width	1 mm	1 week	1.83 ± 1.03	1.60 ± 0.23	0.70
		4 weeks	1.64 ± 0.25	1.26 ± 0.13	0.08
		12 weeks	0.82 ± 0.25	1.13 ± 0.07	0.10
		P value	0.21	0.02*	
	3 mm	1 week	2.19 ± 0.98	1.56 ± 0.20	0.29
		4 weeks	1.38 ± 0.09	1.39 ± 0.08	0.90
		12 weeks	1.33 ± 0.55	1.20 ± 0.08	0.70
		P value	0.26	0.04*	
	5 mm	1 week	2.34 ± 1.11	1.82 ± 0.15	0.42
		4 weeks	1.99 ± 0.20	1.79 ± 0.14	0.24
		12 weeks	2.05 ± 0.31	1.41 ± 0.04	0.07
		P value	0.81	0.008*	
Lingual width	1 mm	1 week	1.26 ± 0.29	1.63 ± 0.26	0.10
		4 weeks	1.36 ± 0.13	1.43 ± 0.04	0.42
		12 weeks	1.20 ± 0.24	1.21 ± 0.36	0.97
		P value	0.72	0.17	
	3 mm	1 week	1.57 ± 0.72	1.92 ± 0.25	0.41
		4 weeks	1.81 ± 0.09	1.95 ± 0.08	0.11
		12 weeks	1.66 ± 0.48	1.55 ± 0.08	0.73
		P value	0.85	0.40	
	5 mm	1 week	1.99 ± 0.57	2.19 ± 0.30	0.56
		4 weeks	2.19 ± 0.03	2.26 ± 0.36	0.79
		12 weeks	2.43 ± 0.46	1.71 ± 0.26	0.08
		P value	0.47	0.12	
Overall BL area	Coronal	1 week	19.68 ± 0.99	17.58 ± 0.34	0.02*
		4 weeks	10.55 ± 0.08	13.68 ± 0.27	< 0.001*
		12 weeks	9.13 ± 0.67	13.02 ± 0.59	0.002*
		P value	< 0.001*	< 0.001*	
	Middle	1 week	23.96 ± 0.55	24.23 ± 0.62	0.53
		4 weeks	14.04 ± 0.78	18.47 ± 0.36	0.001*
		12 weeks	18.50 ± 0.40	16.55 ± 0.43	0.004*
		P value	< 0.001*	< 0.001*	
	Apical	1 week	27.20 ± 0.44	26.44 ± 1.17	0.27
		4 weeks	16.57 ± 1.45	20.37 ± 0.06	0.045*
		12 weeks	21.90 ± 1.11	17.50 ± 0.39	0.003*

T-test was used for comparing the two studied groups, while One-way ANOVA was used for comparing different time points within each group.

*Statistically significant at p value < 0.05.

##### At one week post-operatively (Fig. 4, Table 1 and Supplementary Table 1)

**Fig. 4:**
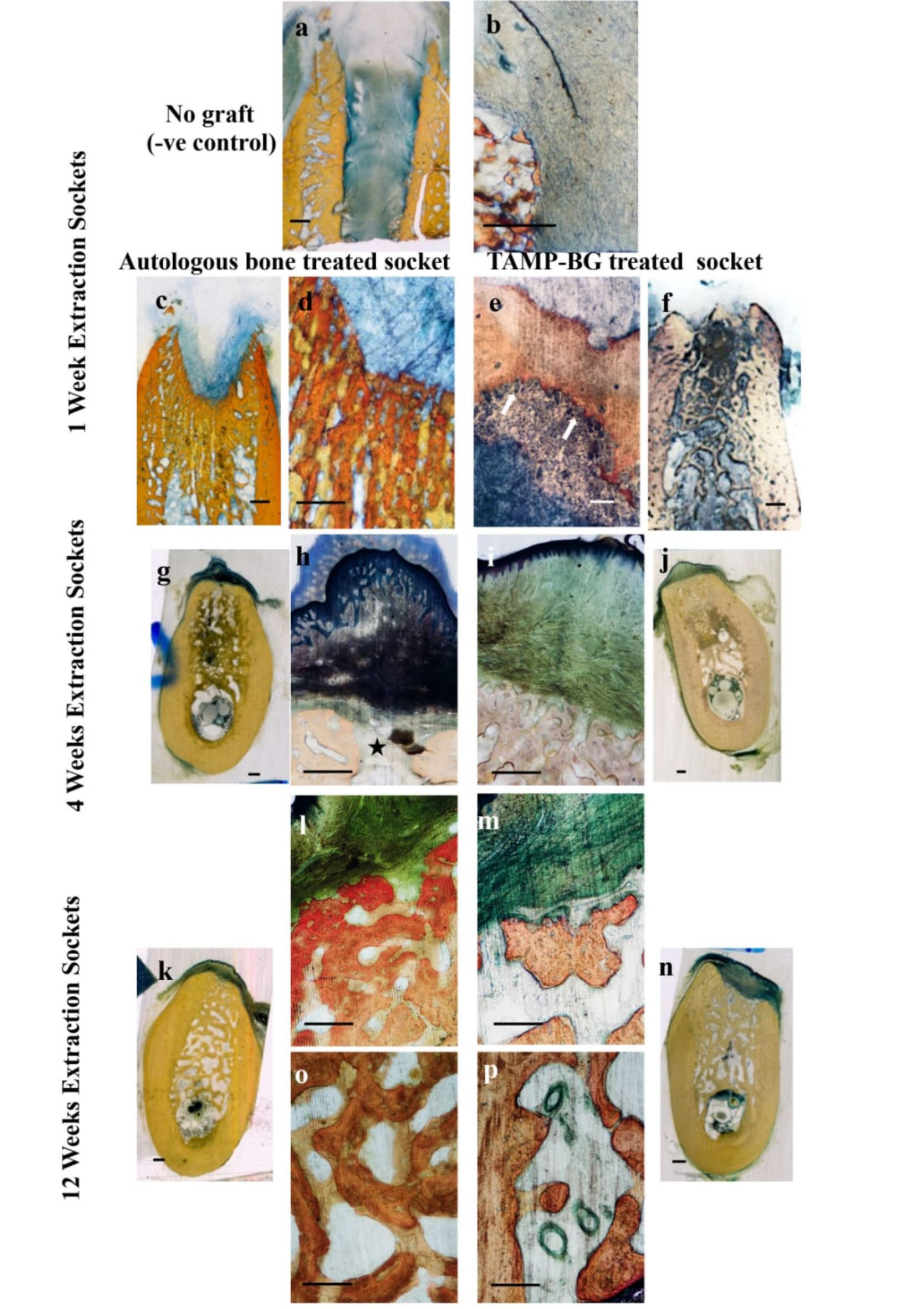
Histological sections cut buccolingually of differently treated ridge specimens, at one week interval. Negative control empty extraction socket (no graft) shows heavily distributed granulation tissue (a, scanned section) with limited signs of regeneration at the side of socket in the form of newly formed bony spicules (b, microscopic appearance of a, apically). (c & d) are showing grafted specimen with autologous bone, where (d) is higher magnification of the scanned section (c), showing regular healing pattern of alveolar socket, with newly formed bone growing from apical to coronal, as the top coronal area was still devoid of bone. Extraction socket that received TAMP-BG in (f) (scanned section) as an overview of the ridge showing newly formed bony spicule-like bands extending in different directions throughout the whole space of the healing socket and demonstrating socket closure by newly formed bony plate (corticalization) one week postoperatively. Microscopic appearance of (f) is shown in (e) with remodeling signs coronally at the bony plate in the form of osteoclastic activity “white arrows” . Remnants of TAMP-BG particles could be noticed (dark blue patches) coronally and between the bony trabeculae (f), with high vascularity in socket central area. At 4 weeks postoperatively (g-j), autologous bone chips treated socket is showing at its most coronal level (h) the microscopic appearance of scanned section in (g) the oral mucosa that has already closed the extraction wound, and overlying a small opening at the alveolar margin and a narrow pathway at the extraction site underneath that was still devoid of bone (black star). While in extraction socket that received TAMP-BG (i & j) complete closure of socket entry could be seen. (i) is the microscopic appearance of the coronal level of scanned specimen in (j), with oral mucosa normally healed, and numerous bone trabeculae with no remnants of TAMP-BG. Comparison of healing between the 2 differently treated sockets are shown up till 12 weeks in k, l & o (autologous bone socket) and m, n & p (TAMP-BG socket), where the contour and form of socket were obviously much more preserved in the experimental socket than those of the control one. A closer look at the coronal and apical levels of the extraction socket that received TAMP-BG (m & p) respectively, which are the microscopic appearance of specimen in (n), demonstrates the same feature and characteristics seen before at shorter intervals; large number of marrow cavities and regenerative signs in the form of thick osteoid tissue lining trabecular surface as well as strong angiogenic activity (p). While the extraction socket treated with autologous bone chips shows reduction in the dimension of the socket (k) and obvious disappearance of the buccal and lingual crests leaving the socket orifice tapered due to bone resorption, denoting a different mechanism of healing as shown coronally and apically (l & o respectively) which are the microscopic appearance of specimen overview in (k). Scale bar on scanned sections equals 1000 µm, that on the 40X microscopic images is 500 µm and 100 µm for 100X (e).

TAMP-BG grafted specimens showed widely distributed newly formed bone trabeculae in all directions; a bony plate covering the socket orifice and granulation tissue underneath with TAMP-BG particles, accompanied by rich angiogenic activity. There was significantly less difference between lingual and buccal crestal heights, compared to that of autologous bone specimens.

##### At 4 and 12 weeks postoperatively (Fig. 4 and Table 1)

At 4 weeks, TAMP-BG treated socket orifice showed full closure. In autologous bone grafted sockets, a small opening at the alveolar margin was still noticeable. After 12 weeks, trabecular bone totally filled both sockets. Marrow areas were widely distributed in TAMP-BG treated specimens, with remarkable cellular and angiogenic activity. At 4 weeks, the TAMP-BG group showed statistically less difference between buccal and lingual crests than that of the autologous bone group; while this was totally reversed at 12 weeks. Moreover, at this same interval, the socket orifice was significantly wider in the TAMP-BG than that grafted with autologous bone.

#### The implant specimens (the distal extraction socket of P4 with grafted implant)

Results of histomorphometric measurements are documented in Table 2 and supplementary Table 2.

**Table 2:** Comparison between the autologous bone and TAMP-BG groups of implant specimens, at different time points, regarding the distance from implant shoulder to 1st BIC, from implant shoulder to bone crest, the difference between buccal and lingual heights and the width of buccal & lingual plates of bone, at 1, 3, 5 mm levels.

			Autologous bone	TAMP-BG	T-test P value
			Mean ± SD		
Shoulder to 1st BIC	Buccal	1 week	4.37 ± 0.27	4.47 ± 0.28	0.64
		4 weeks	3.25 ± 0.11	3.21 ± 0.04	0.49
		8 weeks	3.67 ± 0.13	3.57 ± 0.07	0.26
		P value	< 0.001*	< 0.001*	
	Lingual	1 week	3.79 ± 0.15	4.05 ± 0.17	0.06
		4 weeks	3.50 ± 0.20	2.79 ± 0.42	0.02*
		8 weeks	3.10 ± 0.48	2.97 ± 0.02	0.63
		P value	0.04*	< 0.001*	
Shoulder to bone crest	Buccal	1 week	0.87 ± 0.06	1.33 ± 0.11	0.001*
		4 weeks	2.14 ± 0.30	2.69 ± 0.36	0.06
		8 weeks	3.14 ± 0.03	2.78 ± 0.18	0.007*
		P value	< 0.001*	< 0.001*	
	Lingual	1 week	0.13 ± 0.16	0.95 ± 0.11	< 0.001*
		4 weeks	1.93 ± 0.31	2.41 ± 0.25	0.054
		8 weeks	1.67 ± 0.14	1.20 ± 0.21	0.01*
		P value	< 0.001*	< 0.001*	
L-B crestal height (VD)		1 week	-0.73 ± 0.10	-0.37 ± 0.09	0.002*
		4 weeks	-0.21 ± 0.04	-0.28 ± 0.13	0.38
		8 weeks	-1.48 ± 0.17	-1.58 ± 0.19	0.43
		P value	< 0.001*	< 0.001*	
Buccal width	1 mm	1 week	0.80 ± 0.04	0.77 ± 0.02	0.24
		4 weeks	1.11 ± 0.09	1.12 ± 0.35	0.94
		8 weeks	1.08 ± 0.12	1.06 ± 0.17	0.84
		P value	0.002*	0.11	
	3 mm	1 week	1.80 ± 0.06	1.06 ± 0.14	< 0.001*
		4 weeks	2.64 ± 0.24	1.74 ± 0.52	0.02*
		8 weeks	2.23 ± 0.26	1.76 ± 0.09	0.01*
		P value	0.001*	0.02*	
	5 mm	1 week	2.71 ± 0.15	1.68 ± 0.06	< 0.001*
		4 weeks	3.37 ± 0.51	2.35 ± 0.23	0.02*
		8 weeks	3.55 ± 0.18	2.67 ± 0.13	< 0.001*
		P value	0.01*	< 0.001*	
Lingual width	1 mm	1 week	1.72 ± 0.25	1.46 ± 0.19	0.14
		4 weeks	1.64 ± 0.03	1.66 ± 0.16	0.81
		8 weeks	2.22 ± 0.10	1.91 ± 0.07	0.003*
		P value	0.001*	0.007*	
	3 mm	1 week	3.01 ± 0.53	3.02 ± 0.29	0.99
		4 weeks	3.11 ± 0.32	2.48 ± 0.15	0.02*
		8 weeks	3.13 ± 0.12	2.94 ± 0.19	0.13
		P value	0.89	0.02*	
	5 mm	1 week	3.88 ± 0.35	3.60 ± 0.08	0.21
		4 weeks	4.10 ± 0.23	3.05 ± 0.26	0.001*
		8 weeks	3.59 ± 0.16	3.50 ± 0.09	0.37
		P value	0.06	0.002*	

T-test was used for comparing the two groups, while One-way ANOVA was used for comparing different time points within each group.

*Statistically significant at p value < 0.05.

##### At 1 week postoperatively (Fig. 5, Table 2 and Supplementary Table 2)

Osteogenesis around the implant grafted with TAMP-BG showed the fastest healing and new bone formation (supplementary Fig. 3) with higher invading rate at the implant/bone interface, compared to both control specimens. Degrading TAMP-BG particles could be seen at the implant surface in its middle part (corono-apically). Distance between implant shoulder and the 1^st^ BIC was significantly higher from the buccal side for both TAMP-BG and autologous bone groups than that of the negative control one. From the lingual side; there was no significant difference between the three groups.

##### At 4 and 8 weeks postoperatively (Fig. 5 and Table 2)

**Fig. 5:**
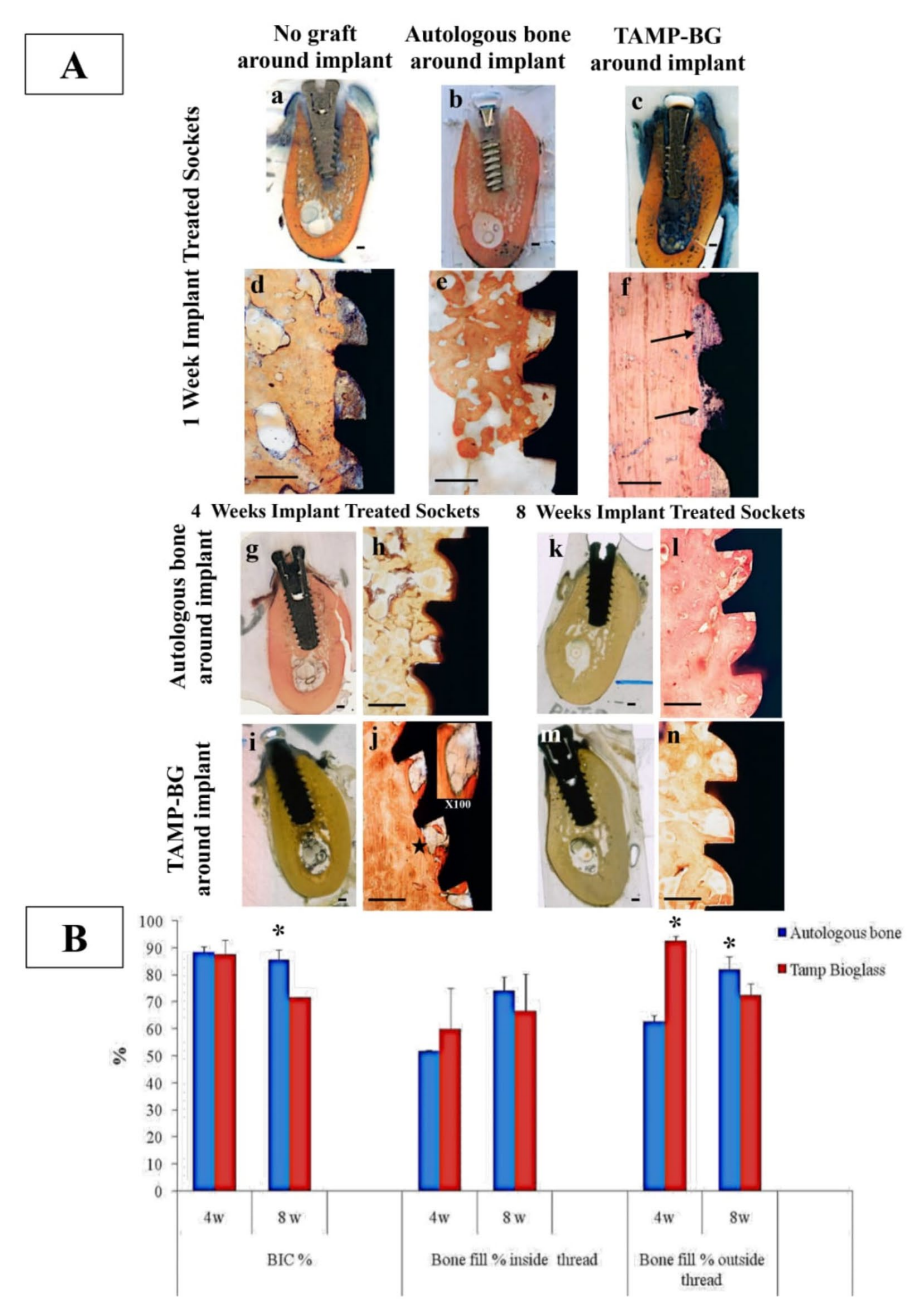
Panel (A) shows histological sections cut bucco-lingually of differently treated immediate implants in mandibular extraction sockets at one week interval. (**a**), (**b**) & (**c**) are the scanned sections of microphotographs (**d**) (no graft around implant), (**e**) (socket received autologous bone around implant) & (**f**) (TAMP-BG around implant), respectively. Variable rates of new bone formation could be noticed at the bone/implant interface, when the 3 specimens are compared, where the least degree of osseointegration is shown in the socket that received the un-grafted implant (**d**), while the highest one is observed in the socket that received TAMP-BG around implant (**f**). Few remnants of TAMP-BG material are still noticeable {arrows} in (**f**) at that time interval around surface serrations of implant. At 4 weeks interval following implant insertion, different osseointegration mechanisms were observed at the bone/implant interface in TAMP-BG treated socket (**i** & at higher magnification in **j**), when compared to socket that received autologous bone around implant (**g** & at higher magnification in **h**). Large marrow cavities (black star) lined completely all around at the border by secretory osteoblasts and reaching the implant surface, demonstrating highly active spots of newly formed bone at the bone/implant interface, with secretory cells lining both bony socket wall as well as extending in a circle like form to line the implant surface (insert at top right of j (100X). No BG remnants could be seen at this time interval. While autologous bone treated socket (**h**) has shown less bone density with wider un-mineralized matrix area than that treated with TAMP-BG (**j**). Consistently, difference in bone pattern regeneration at implant/bone interface could still be observed 8 weeks post implantation, between the socket that received autologous bone graft around implant (**k** & at higher magnification in l) and that with TAMP-BG (**m** & at higher magnification in n). New bone in TAMP-BG implanted socket maintained a rich distribution and highly organized wide marrow spaces just along implant/bone interface (**n**), with angiogenesis and cellular lining, reflecting the unique mechanism of osseointegration as a cascade of degradation of the bioactive TAMP-BG, confirming the same characteristic pattern of bone regeneration at shorter interval of the same group. While the control healed socket (**l**) is showing the classic appearance of osseointegrated implant with no marrow cavities, yet dense bone at the interface. Scale bar on scanned sections equals 1000 µm and that on the 40X microscopic images is 500 µm. Panel (B) shows Graphical presentation of histomorphometric analysis of differently treated implant specimens; representing the BIC %, bone fill % inside and outside implant threads. All data represent means and standard deviations. Statistically significant differences between the test and control groups are demonstrated by the (*) on top of columns.

At 4 weeks, bone at the implant/bone interface in TAMP-BG specimens, grew at the socket wall as well as at the implant surface, bridged by secretory osteoblasts, while in autologous bone/implant treated sockets, bone originated from the socket wall. Bone fill% outside the threads for TAMP-BG grafted sockets recorded significantly higher value (92.71% ± 1.71), with thicker trabeculae than that for autologous bone sockets (62.92% ± 2.02) (P = 0.0001).

At 8 weeks, TAMP-BG treated implants demonstrated more organized distribution of marrow tissue compared to that of autologous bone grafted sockets. Bone fill% outside the threads was significantly higher in autologous bone group (82.01% ± 4.87) compared to that of TAMP-BG group (72.74% ± 3.91) (P = 0.0250).

Dynamic osseointegration in the TAMP-BG sockets was noticeable, as bone deposited on implant serrations was like the trabecular pattern of TAMP-BG (Supplementary Fig. 1). Both groups showed remarkable osseointegration as early as 4 weeks, recording (87.70% ± 5.16) for TAMP-BG group, and (88.46% ± 1.99) for autologous bone group. However, at 8 weeks autologous bone treated implant had a significantly higher BIC% (85.81% ± 3.46) than that of TAMP-BG group (71.66% ± 0.17) (P = 0.0002). On the other hand, TAMP-BG grafted socket showed significantly shorter distance between the implant shoulder to the 1^st^BIC, lingually.

Measurements of difference between buccal and lingual crestal heights showed reduction in values from 1 to 4 weeks, but then values drastically increased from 4 to 8 weeks for both groups, with a consistently shorter buccal plate than the lingual one.

For the TAMP-BG group, the buccal width plate showed progressive increase significantly from 1 to 8 weeks at 3 mm level. Regarding the 5 mm level, the autologous bone group showed significantly wider plate at all intervals than that of TAMP-BG group.

Concerning the lingual plate width values at 1 mm level, 8 weeks autologous bone showed significantly wider plate than that of TAMP-BG group, while TAMP-BG group showed significantly progressive increase in width from 1 to 8 weeks. At 3 & 5 mm levels, autologous bone group showed significantly wider plate than TAMP-BG group at 4 weeks only.

## Discussion

The current study focused on using a characterized bioactive glass scaffold with multiscale porosity (TAMP-BG) for alveolar ridge augmentation and reconstruction of bone defects following tooth extraction for immediate implant placement. Although guided bone regeneration (GBR) is a well-established surgical procedure used to construct bone defects around dental implants, its value in relation to immediate implant placement has been challenged^24^.

A recent metanalyses concluded that there were minimal differences in crestal bone loss around immediately placed implants with or without GBR. However, the authors added that the effects of GBR maybe more important in larger versus smaller bone defects. Additionally, the crestal bone loss changes around immediately placed implants with GBR compared with conventional implant placement were minimal although they might be esthetically important. This has encouraged further research for more regenerative techniques using different bone substitute materials for bone defects reconstruction around immediately placed implants. Although pooled analyses and critical appraisals regarding this topic are still lacking^25^. Current evidence points to a reduction of the amount of horizontal buccal resorption and an improvement of long-term peri-implant soft tissue esthetics compared to implants not receiving bone substitute materials. These controversies and lack of high quality of evidence were the driving forces behind the rationale of the current study.

Regarding the initial degradation stage of TAMP-BG scaffold, it was characterized by increase in the pH due to fast release of calcium ions as a function of increase in the surface area leading to alkaline environment. This sequence of events started at 1^st^ week and continued till the 4^th^ week, as demonstrated by SEM/EDX, and was confirmed by FTIR and XRD.

The formation of calcium phosphate layer retarded further dissolution process that enabled the scaffold to maintain the tissue space until complete tissue regeneration, while the surface showed greater roughness that provided more nucleation sites for calcium phosphate crystallization gradually, that was indicated from the 2nd week of immersion.

Implanting the TAMP-BG scaffold under rabbit skin for seven days revealed its faster degradation under this dynamic environment, while scaffold surface became covered with the bioactive HCA layer that enhanced cell attachment***.***

In the present study, the proliferation effect of cultured medium supplemented with TAMP-BG extract on hABMSCs obtained from human alveolar bone marrow aspirate was coupled with enhanced cell migration, which appeared from the MTT assay up to 5 days, and then it decreased afterward**.**

Recent information reported that Si could stimulate not only the migration and differentiation of healthy cells, but also enhanced the apoptosis of injured cells, by increasing the cell membrane fluidity^26^. This may explain the decrease in the proliferation of cultured cells regardless the concentration of TAMP-BG extract in the media. Increased cell membrane fluidity produces a wide range of biological activities e.g. cell migration, proliferation and differentiation^26,27^. This has been suggested to be the result of upregulation of key signaling pathways such as those related to G protein, cytokine receptors, and focal adhesion. Furthermore, increased extracellular calcium ions have been shown to trigger remarkable elevation of osteopontin production from mesenchymal stem cells leading to further induction of cell recruitment in the target microenvironment for tissue regeneration^28^.

The benefit of maintaining the specific 3/D geometry of the TAMP-BG pieces was to keep the scaffolds in position for at least 4 weeks or more, while enhancing the degradation, in order to promote bone regeneration within the dimension of the extracted socket, as was demonstrated under the skin. We believe that the highly dynamic environment of the extracted socket allowed continuous fluid transport to dampen the pH rise. At the earlier stage of healing, our histological findings showed that the healing pattern in sockets grafted with cancellous bone autograft did not differ in general from non-grafted sockets, indicating few bony spicules growing from apical and lateral compartments. A void was confined in the central zone of the coronal and middle regions, in continuity with the entrance of the alveoli. This result was in agreement with many investigators^5,29,30^.

Healing of sockets grafted with TAMP-BG was different than the other two groups. TAMP-BG sockets showed obvious corticalization process; hard tissue bridge covering the socket entrance thus keeping the level of the buccal crest more coronally than the lingual crest of the socket walls.

Bucco-lingual dimension of socket coronally was significantly greater in autologous bone socket than that of TAMP-BG one. This has been previously reported, due to the slow degradation of bone chips, compared to that of the synthetic one, at this early phase of healing^5,6,31^***.***

In the specimens of TAMP-BG grafted group, the pattern of new bone formation at the implant threads surface extended in different directions due to enhanced wettability and osteoblasts attachment, modulated by the 3/D scaffold architecture.

By 4 weeks, the progress of healing of auto-graft sockets in the present research was in agreement with results reported by many investigators^5,32^***,*** indicating that the grafted bony chips failed to preserve ridge dimensions; as reflected in the loss of buccal and lingual walls height of the socket and the reduction of bucco-lingual dimension, with obvious incomplete closure of hard tissue cap coronally.

Although, the degradation process left the trabecular branches of TAMP-BG scaffold thinner, yet we believe that the 3/D nature of the particles of synthetic graft maintained the horizontal dimension of the alveolar socket and left the buccal and lingual crests height almost at the same level. Interestingly, these features continued till 3 months, showing horizontal ridge preservation at the coronal third of the extraction socket that developed better contour required for implant placement, compared to that of autologous bone socket. However, the progressive reduction of the buccal bony plate by 12 weeks of sockets treated with TAMP-BG could be attributed to the complete degradation and disappearance of the scaffold by the end of this interval.

A recent systematic review confirmed that one of the essential scaffold criteria is to maintain the volume of the defect for a sufficient time until it is completely replaced by new bone tissue^33^***.*** We observed the unique pattern of TAMP-BG degradation that resembled natural trabecular bone resorption, preserving the volume for 12 weeks.

Osseointegration started from 1st week where TAMP-BG was grafted around dental implant***,*** as it was influenced by Ca and Si ions release in the environment, that represented the key elements in recruitment, proliferation and differentiation of mesenchymal stem cells^26,28,34^***.***This successful osseointegration that was observed histologically in the current work was recently proven to enhance mechanical stability with Ca coated implants^35^.We did not observe this phenomenon in the two other studied groups at the same interval.

The influence of bioglass dissolution products on macrophage responses was recently highlighted and has been demonstrated to be crucial for tissue construction through modulating osteogenesis, osteoclastogenesis, and angiogenesis^34,36^***.*** Indeed, bioactive glasses have been shown to exert immunomodulatory effects through the activation of a cascade of events at the bone injury site by switching between macrophage phenotype M1 (pro-inflammatory) and M2 (anti-inflammatory) which favors the bone regenerative pathway. Elevated calcium ions have also been shown to stimulate the secretion of BMP-2 by macrophages^34^. Macrophage-derived IL-10 can further regulate the osteogenic markers such as collagen type I alpha 1 (COL1A1), alkaline phosphatase, and RUNX-2 elucidating the importance of the immune system in bone regeneration^37^.This is supported by our findings that recorded the presence of osteoclast-like cells at the first two weeks in TAMP-BG grafted sockets, and around implants on the bone ridges opposing implant surfaces, next to osteogenic cells. However, the exact mechanisms and pathways behind the role of the immune system in bone regeneration remain elusive. In the present study, the standardized preparation process of our tailored bioactive glass regarding its chemical composition, porosity pattern, controlled release of bioactive ions, and their impact in the biological microenvironment were reproducible as evidenced by the extensive characterization tests to ensure the regenerative efficacy of TAMP-BG. Additionally, the preparation technique used in this study was previously validated in our precedent publications^21,22^.

The dynamic microenvironment around implants grafted with TAMP-BG continued up to 8 weeks, showing new bone deposited on the surface of the implant serration, yet maintaining the large bone marrow spaces. The obvious difference in the way of osseointegration between implants grafted with autologous bone versus implants grafted with TAMP-BG reflected the influence of biomaterial parameters at the implant/bone interface and opposing bone surface. The bone deposited on the surface of the implant serrations followed the same pattern of the 3/D architecture of the tailored scaffold. Nano/macro porous architecture of bone deposition on the implant surface occupied empty spaces that enhanced implant fixation to the bone^38^.Our results showed progressive increase in the buccal bone plate width around TAMP-BG implant over time from 1 to 8 weeks, at 3and 5mm levels, reflecting the dynamic cellular activity in the microenvironment***.***

Although the distance between implant shoulder and alveolar bone crest was longer for the TAMP-BG group than that of autologous bone group, from both sides at 4 weeks, the situation was reversed by 8 weeks; demonstrating new bone formation in the vertical direction in TAMP-BG sockets. This was one of the important findings in this work, confirming the long-term effect of the TAMP- BG scaffold, even after complete degradation, which was not reported by previous investigators.

As we discuss the promising effects of TAMP-BG in bone regeneration in ridge and around implants sites, we acknowledge the limitations of our study. These include small number of investigated animals, short survival periods, and lack of data regarding effect of TAMP-BG on soft tissue healing around implants. For future studies, we recommend the investigation of different forms of scaffold architecture as well as chemical compositions that affect the bioactive material characteristics. We also recommend the use of microtomographic analysis of the regions evaluated by histomorphometric analysis to evaluate the entire area of interest and the implementation of specific signaling pathways to assess how TAMP-BG promotes the proliferation and migration of hABMSCs. Concerning the clinical impact, alveolar ridge preservation is crucial for immediate implant placement. TAMP-BG is a tailored bioactive scaffold that can be used for “personalized” tissue engineering approaches. TAMP-BG can be used as a bioactive dynamic scaffold to prevent socket collapse and preserve the alveolar ridge following tooth extraction and around immediately placed dental implants.

## Conclusions

Bioactive glass scaffolds can act as carriers for controlled release of therapeutic ions like silicon and calcium, as long as the degradation continues. One of the essential scaffold criteria is to maintain the volume of the defect for a sufficient time to match the tissue growth rate. It is possible to modify the scaffold physico-chemical properties, by fine tuning the processing parameters, in terms of its porous structure, surface area, type and rate of ions release, in order to control degradation time, and enhance its effect on alveolar bone preservation and new formation around immediately placed implants.

## Methods

### TAMP-BG preparation and characterization

Tailored amorphous multiporous (TAMP-BG) scaffold (70 mol% SiO2/30 mol% CaO2) was prepared by a modified sol–gel process as previously described^22^.

### Chemical characterization of TAMP bioactive glass

#### Bioactivity and dissolution studies

Bioactivity was investigated to observe the formation of hydroxyl-carbonate apatite (HCA) on the surface of the prepared TAMP-BG samples for 1, 2, 3, and 4 weeks^23^. PBS extracts were used to determine the (Si) and (Ca) concentrations and the pH values. The discs were then characterized by scanning electron microscopy (SEM JEOL JSM 6360LA, Japan) with energy dispersive x-ray spectrometry (EDX). Both Fourier Transform– IR Spectrophotometer (FTIR -8400S, Shimadzu, Japan) and x-ray diffraction (XRD) scans using (X-ray 7000 Shimadzu, Japan), were used to detect the conformational and crystallographic changes on the surface of the bioactive glass samples.

### Biological characterization of TAMP-BG: effect of TAMP-BG extract on migration and proliferation of human alveolar bone marrow-derived mesenchymal stem cells (h-ABMSCs)

#### Preparation and analysis of TAMP-BG extract

TAMP-BG powder was soaked in serum free Dulbecco’s modified Eagle’s medium (DMEM) *(Lonza, Verviers, Belgium)* at a concentration of 0.01 g/mL. The bioactive glass extract was freshly prepared before each experiment^39,40^. The extract was analyzed using inductive coupled plasma atomic emission spectroscopy (*5100* 14 *ICP-OES) (Agilent technologies, California, U.S.A)* Preparation and quality control were carried out according to standard methods^41^.

#### Culture of “hABMSCs”

Mesenchymal stem cells used in this study were derived from human alveolar bone marrow aspirate as previously described^42,43^. Cells from passages 3–5 were used for all experiments.

#### Cell proliferation assays using MTT

The effect of the bioactive glass extract on proliferation of hABMSCs was assessed using MTT cell viability assay (n = 3, in triplicate). Cells were incubated in complete medium for 24 h, and then in serum free media overnight. The cells were then incubated with one of the following conditions: serum free media, 10%FBS, 2%FBS, 100%TAMP-BG extract + 2%FBS and 50%TAMP-BG extract + 2%FBS. Cell proliferation was evaluated after 24, 48,120 and 144 h.^44^.

#### Wound scratch migration assays

The wound scratch assay was used to assess the effect of ionic dissolution products of TAMP-BG on the migration of hABMSCs 3 times in quadruplicate. Cells were plated and upon confluence, scratches were made using P200 pipette tips. The same conditions as for the proliferation assays were used. The rate of wound closure was assessed at 0, 24 and 48 h^45^***.***

### Animal surgery (Supplementary Fig. 6)

Mongrel dogs were selected as a model to develop this study due to their availability as a canine strain in Egypt and because they have been used widely for periodontal regeneration and peri-implant defect research due to anatomical and physiological similarities with humans. Twenty-two healthy male mongrel dogs of 15 to 20 kg and aged 7 to 12 months were used in this study following approval of the institutional ethics committee of Alexandria University; Institutional Animal Care and Use Committee “ALEXU-IACUC” and according to the ARRIVE guidelines (supplementary file). All experimental animal procedures were done in accordance with institutional guidelines and regulations of Alexandria University. Healthy animals with good oral health were obtained from the animal unit of the faculty of veterinary medicine, Alexandria University.

Animals were kept for adaptation for 1 week prior to surgical procedures. Anesthesia was obtained using IM administration of Xylazine HCL (1 mg/kg) *(Xylaject, Adwia, Egypt)* followed by IV administration of Ketamine HCL (10 mg/kg) *(Ketamine, Sigma).* Atraumatic extractions of the mandibular fourth premolar tooth on both sides were done. On the right side, 1mm^3^ prewetted TAMP-BG particles were applied around the immediately placed 8 × 3.7 Ti implant *(SwissPlus, Zimmer, Germany)* thread surface in the distal socket before full screwing of the implant, while the left side received autologous bone chips taken from the iliac crest of the same animal after being cut into almost 1mm^3^ sized pieces and moistened with saline. The extraction socket of the mesial root of the same tooth received TAMP-BG on the right side of the mandible, while autologous bone was applied on the left side. Each socket was filled with the grafting material until complete flushing with the gingival margin. Right side sockets received 0.3538 to 0.729 g of TAMP-BG. The implant was placed by the same surgeon in the osteotomy site taking care not to be inserted to the full length. Bone graft particles were applied to the threads of the implants prior to be screwed to the full length so the bone graft particles filled the gap between the implant and the extraction socket wall.

Three interrupted sutures were taken using 4/0 resorbable glycolide-Poly-L-lactide material *(Vicryl, Ethicon,USA)*. Following surgery, animals received intramuscular Cephotaxime1g antibiotic (Egyptian. Int. Pharmaceutical (E.I.P.I.C.O),10^th^ of Ramadan, Egypt) in a dose of 150 mg/Kg/day and Ketorolac analgesic (Amriya Pharm. IND, Alexandria, Egypt) 60 mg/day for 3 days. Animals were euthanized at 1, 2, 4, 8 & 12 weeks post-operatively using an overdose administration of thiopental sodium (Amriya Pharm. IND, Alexandria, Egypt) (Fig. 1A).

### Histological sample preparation

Specimens were processed for non-decalcified hard sectioning using plastic resin embedding technique as previously described^46^***.*** Specimens were cut longitudinally in a bucco-lingual plane into serial Sects. (150 μm) using (EXAKT band system 300 CP, EXAKT technologies, USA). Sections were stained with Stevenel’s blue and Van Gieson (Sigma-Aldrich); both are specialized stains used in resin embedding technique of hard tissues for their effectiveness in demarcation between soft and hard structures. Sections were then examined under light microscope (Olympus CH40RF200, Japan) by a single blinded expert investigator following calibration for intraexaminer reliability to minimize bias. Evaluation was done in the 3 serial sections in the central part of the defect with 100 microns apart. In the implant specimens, the entire length of implant (from shoulder to base of implant) was used to ensure consistency in evaluating the center of the defect.

### Histomorphometric analysis for ridge and implant specimens (Fig. 1B)

For ridge specimens; difference between buccal and lingual heights, coronal contour, lingual and buccal plates’ widths at 1, 3& 5 mm levels^29^; and total bucco-lingual area at coronal, middle and apical thirds of the ridge^47^ were measured. In implant specimens, distance from implant shoulder to 1^st^ bone implant contact (BIC) point on both buccal and lingual sides, distance from implant shoulder to the lingual and buccal bone crests, difference between buccal and lingual heights^48^***,*** lingual and buccal plates’ widths at 1, 3 & 5 mm levels, bone implant contact (BIC) % and bone fill % inside and outside implant threads^49^, were measured***.*** Bone fill % within the implant threads; represented the new bone regenerated in close contact with the implant surface throughout its whole length. This was done first by tracing the whole area confined between each two threads, and then bone areas within the first traced area were measured to be divided by total area. Whereas, bone fill % outside the threads area was done in a rectangular area lateral to the threads all along the implant’s length from the first most coronal implant thread to the implant base.

All parameters were measured on scanned histological sections (*HP Scanjet G3010 scanner with resolution of 1200 pixels/inch)* using image J software^50^ except bone fill % parameters which were calculated on microscopic images.

### Statistical analysis

Data were analyzed using IBM SPSS for Windows (Version 23.0). Mean and standard deviation (SD) were calculated. Comparisons between two control groups and TAMP-BG groups at one week and between different time points within same groups were done using one-way ANOVA, followed by multiple pairwise comparisons using Bonferroni adjusted significance levels. At 4, 8 and 12 weeks comparisons between autologous and TAMP-BG groups were done using independent samples t-test. Data of BIC % and bone fill % were analyzed using Graph pad software for Windows (Version 10.3.0), California, USA) using the unpaired student’s t-test for means and standard deviations. Significance for all tests were considered at p < 0.05.

## Electronic supplementary material

Below is the link to the electronic supplementary material.

Supplementary Material 1.

## Data Availability

Data is provided within the manuscript or supplementary information files.
