# Supplementary material for: Tailoring and characterization of bioactive graft material for alveolar bone preservation and regeneration in fresh extraction sockets of dog model
Source: Sci Rep. 2025 Jan 27;15:3321. doi: 10.1038/s41598-025-86408-x (PMC11770185; doi:10.1038/s41598-025-86408-x)
Supplement: Supplementary file 1 — Supplementary Material 1. [file 41598_2025_86408_MOESM1_ESM.docx]

**Scanning electron microscope (SEM) evaluation of the bone/implant interface:**

Four animals were sacrificed; two at 2 week and two at 4 week intervals. The right and left sides of the mandible were separated, and the regions that received the implants were trimmed leaving 0.5 cm from the jaw around the area of interest. The samples were fixed in 2% paraformaldehyde and 2.5% glutaraldehyde in 0.1*M* Na-cacodylate buffer(pH7.3–7.4) “*Electron Microscopy Sciences”*, fixative for3 days. Samples were washed three times in 0.1*M* Na-cacodylate buffer (pH 7.3–7.4), and then freeze-fractured in liquid nitrogen for 2-3 minutes. Using a chisel and a rubber mallet, the specimens were split into two halves, one containing the implant embedded in bone and the corresponding bone sample detached from the implant(Davies & Baldan, 1997).Bone samples were then fixed in 1% *Osmium tetroxide “Sigma-Aldrich”* in 0.1*M* Na-cacodylate buffer (pH 7.3–7.4) for one hour. The samples were then dehydrated in graded concentrations of ethanol (50%, 70% and 100%); one day incubation for each concentration, and they were left for 24 h for air drying. The specimens were then coated with gold for scanning *(SEM JEOL JSM 6360LA", Japan)* examination.

**References**

Davies, J. E., & Baldan, N. Scanning electron microscopy of the bone-bioactive implant interface. *J Biomed Mater Res (1997) 36*(4), 429-440.

|  | | **Negative control** | **Autologous bone** | **TAMP-Bioglass** | **One-way ANOVA**  **P value** |
| --- | --- | --- | --- | --- | --- |
|  |  | **Mean ± SD** | | |  |
| **L-B crestal height** | | **-0.25 ± 0.14^a,b^** | **-0.71 ± 0.26^b^** | **0.12 ± 0.45^a^** | **0.01*** |
| **Coronal contour (orifice)** | | **3.82 ± 0.84** | **4.70 ± 0.15** | **3.97 ± 0.33** | **0.09** |
| **Buccal width** | **1mm** | **0.55 ± 0.10^a^** | **1.83 ± 1.03^b^** | **1.60 ± 0.23 ^a,b^** | **0.03*** |
|  | **3mm** | **1.71 ± 0.14** | **2.19 ± 0.98** | **1.56 ± 0.20** | **0.33** |
|  | **5mm** | **2.42 ± 0.36** | **2.34 ± 1.11** | **1.82 ± 0.15** | **0.43** |
| **Lingual width** | **1mm** | **1.05 ± 0.29^a^** | **1.26 ± 0.29 ^a,b^** | **1.63 ± 0.26^b^** | **0.04*** |
|  | **3mm** | **1.77 ± 0.26** | **1.57 ± 0.72** | **1.92 ± 0.25** | **0.57** |
|  | **5mm** | **2.53 ± 0.37** | **1.99 ± 0.57** | **2.19 ± 0.30** | **0.25** |
| **Overall BL area** | **Coronal** | **21.69 ± 0.13^a^** | **19.68 ± 0.99^b^** | **17.58 ± 0.34^c^** | **<0.001*** |
|  | **Middle** | **25.64 ± 0.24^a^** | **23.96 ± 0.55^b^** | **24.23 ± 0.62^b^** | **0.002*** |
|  | **Apical** | **25.59 ± 0.37^a^** | **27.20 ± 0.44^b^** | **26.44 ± 1.17 ^a,b^** | **0.04*** |

**Supplementary Table 1**

Comparison of ridge measurements between the three differently treated ridge specimens, after one week; regarding the difference between buccal and lingual heights, the coronal contour (linear measurement at the orifice of the socket), the buccal and lingual plates widths at 1, 3 & 5 mm levels and the overall bucco-lingual coronal, middle and apical areas.

*Statistically significant at p value < 0.05.

^a, b, c^: different letters denote statistically significant difference between groups using Bonferroni adjusted significance level .

|  | | **Negative control** | **Autologous bone** | **TAMP-Bioglass** | **One-way ANOVA**  **P value** |
| --- | --- | --- | --- | --- | --- |
|  |  | **Mean ± SD** | | |  |
| **Shoulder to 1st BIC** | **Buccal** | **3.19 ± 0.18^a^** | **4.37 ± 0.27^b^** | **4.47 ± 0.28^b^** | **<0.001*** |
|  | **Lingual** | **4.19 ± 1.14** | **3.79 ± 0.15** | **4.05 ± 0.17** | **0.70** |
| **Shoulder to bone crest** | **Buccal** | **0.80 ± 0.02^a^** | **0.87 ± 0.06^a^** | **1.33 ± 0.11^b^** | **<0.001*** |
|  | **Lingual** | **0.55 ± 0.15^a^** | **0.13 ± 0.16^b^** | **0.95 ± 0.11^c^** | **<0.001*** |
| **B-l crestal height (VD)** | | **-0.25 ± 0.14^a^** | **-0.73 ± 0.10^b^** | **-0.37 ± 0.09^a^** | **<0.001*** |
| **Buccal width** | **1mm** | **0.63 ± 0.12^a^** | **0.80 ± 0.04^b^** | **0.77 ± 0.02 ^a,b^** | **0.03*** |
|  | **3mm** | **1.62 ± 0.11^a^** | **1.80 ± 0.06^a^** | **1.06 ± 0.14^b^** | **<0.001*** |
|  | **5mm** | **2.55 ± 0.29^a^** | **2.71 ± 0.15^a^** | **1.68 ± 0.06^b^** | **<0.001*** |
| **Lingual width** | **1mm** | **1.38 ± 0.13** | **1.72 ± 0.25** | **1.46 ± 0.19** | **0.08** |
|  | **3mm** | **2.63 ± 0.26** | **3.01 ± 0.53** | **3.02 ± 0.29** | **0.30** |
|  | **5mm** | **3.38 ± 0.28** | **3.88 ± 0.35** | **3.60 ± 0.08** | **0.08** |

**Supplementary Table 2**

Comparison of extracted sockets measurements between the three differently treated implant specimens, after one week, regarding the distance from implant shoulder to 1st BIC, from implant shoulder to bone crest, the difference between buccal and lingual heights, and the buccal and lingual plates widths at 1, 3 & 5 mm levels.

*Statistically significant at p value < 0.05.

^a, b, c^: different letters denote statistically significant difference between groups using Bonferroni adjusted significance level

**Supplementary figure 1:**

**
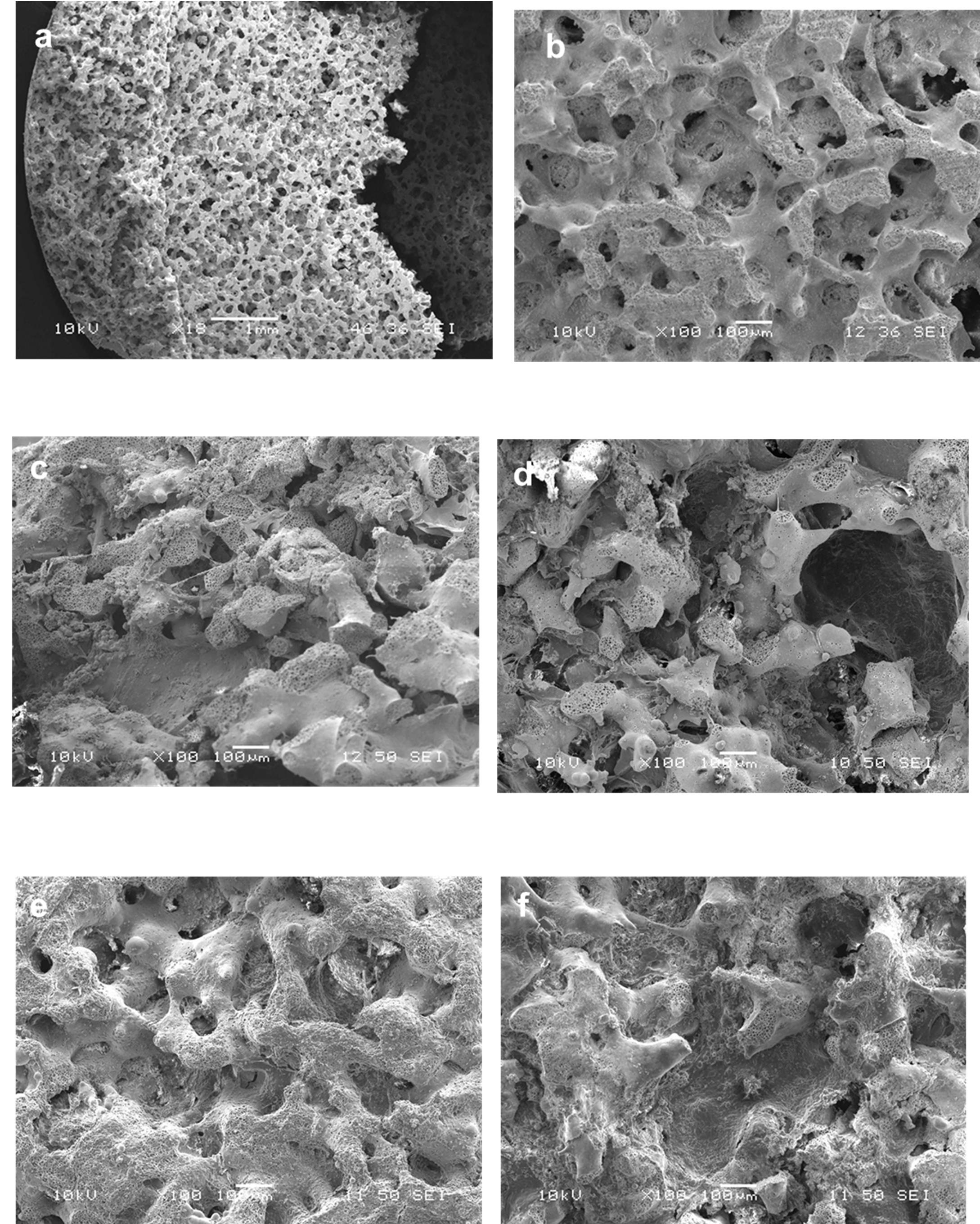
**

**Supplementary figure 1:**

Showing SEM imaging of TAMP-BG specimens implanted under rabbit skin to assess in vivo biodegradation.

a- TAMP-BG disc showing its highly amorphous porous nature SEM X18.b- TAMP-BG disc showing the nano-macro porous structure within the trabecular structure SEM X100. c- Implantation of TAMP-BG disc under rabbit’s skin for one day showing the HCA layer, covering almost 50% of the porous trabecular branches of the scaffold. Numerous Cells appeared to attach between the trabecular branches SEM X100.d- Bioglass disc After 3 days of TAMP-BG disc implantation more surface of the porous scaffold covered with the HCA with Obvious degradation of the scaffold indicated by the broken branches of the trabecular structure SEM X100.e- After 5 days of implantation showing complete covering of the scaffold surface with the HCA layer SEM X100.f- After 7 days of implantation showing continuing degradation and breaking down of the trabecular structure. Obvious porosity still existed underneath the HCA bioactive layer in the remaining parts of the scaffold SEM X 100.

**Supplementary figure 2:**

:
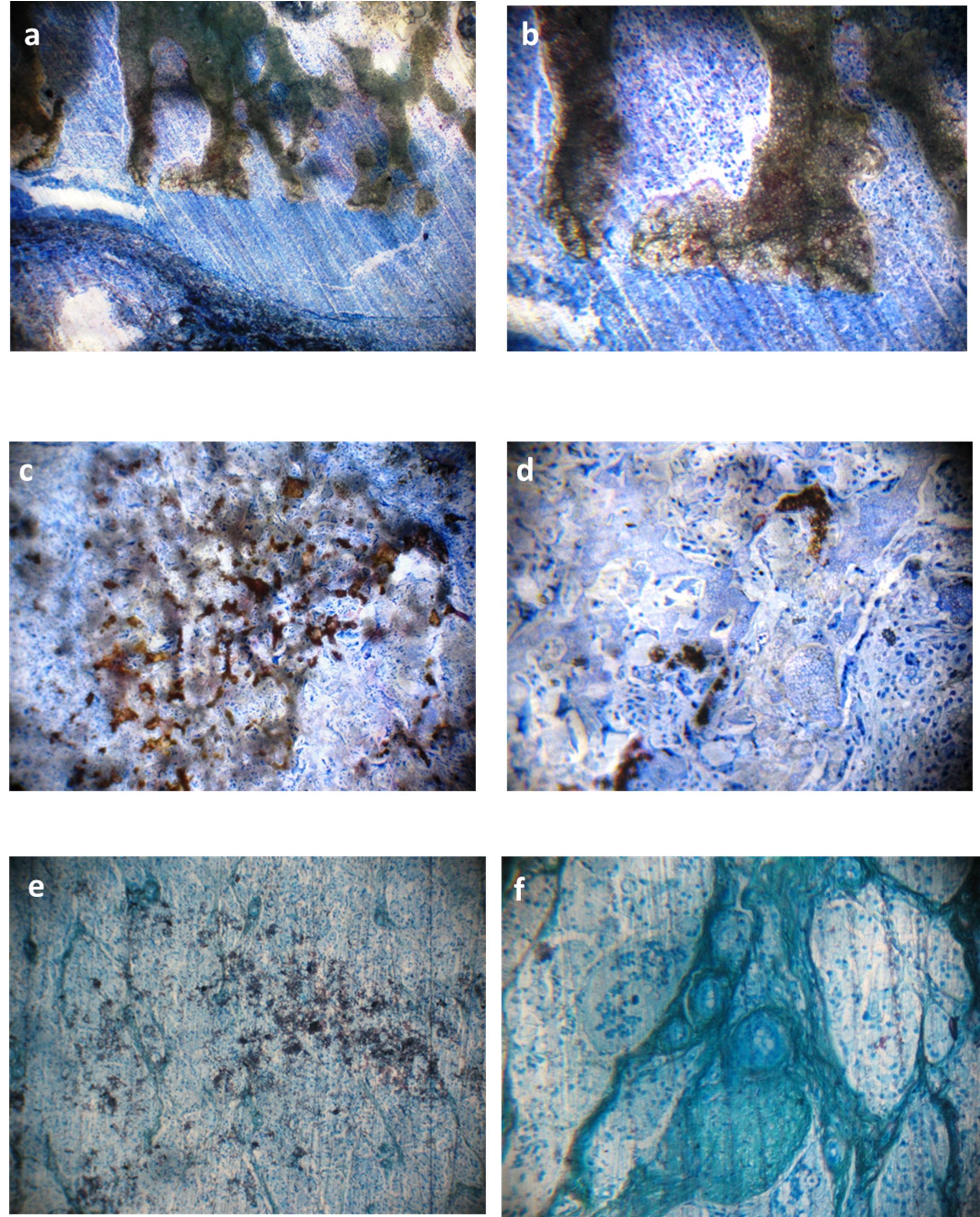


**Supplementary figure 2:**

Showing histological pictures of TAMP-BG implanted under rabbit skin. This experiment was performed to study the degradation behavior of TAMP in-vivo which was previously demonstrated to start one week post implantation .a- 3/D TAMP-BG disc implanted under rabbit skin for one week showing branching pattern of porous scaffold, high inflammatory cells invasion & capsule formation (Original magnification (OM) X4).b- Higher magnification of (a) showing the macro porosity of TAMP-BG scaffold (OM X10).c- After 4 weeks of implantation; Obvious thinning of scaffold branches due to degradation & dissolution, with preservation of 3/D architecture (OM X4).d- Higher magnification of previous scaffold at 4 weeks interval. The remaining degradable scaffold is surrounded by multinucleated cells (OM x10). e- At 8 weeks intervals intense degradation of the scaffold, obvious large blood vessels without any signs of inflammatory cells invasion were seen (OM X4).f- Higher magnification of the previous specimen at 8 weeks showing organized collagen strands, the degraded remnants of scaffold were not seen at this magnification, instead large blood vessels existed between the collagen fibers strands (OM X10).

**Supplementary figure 3:**


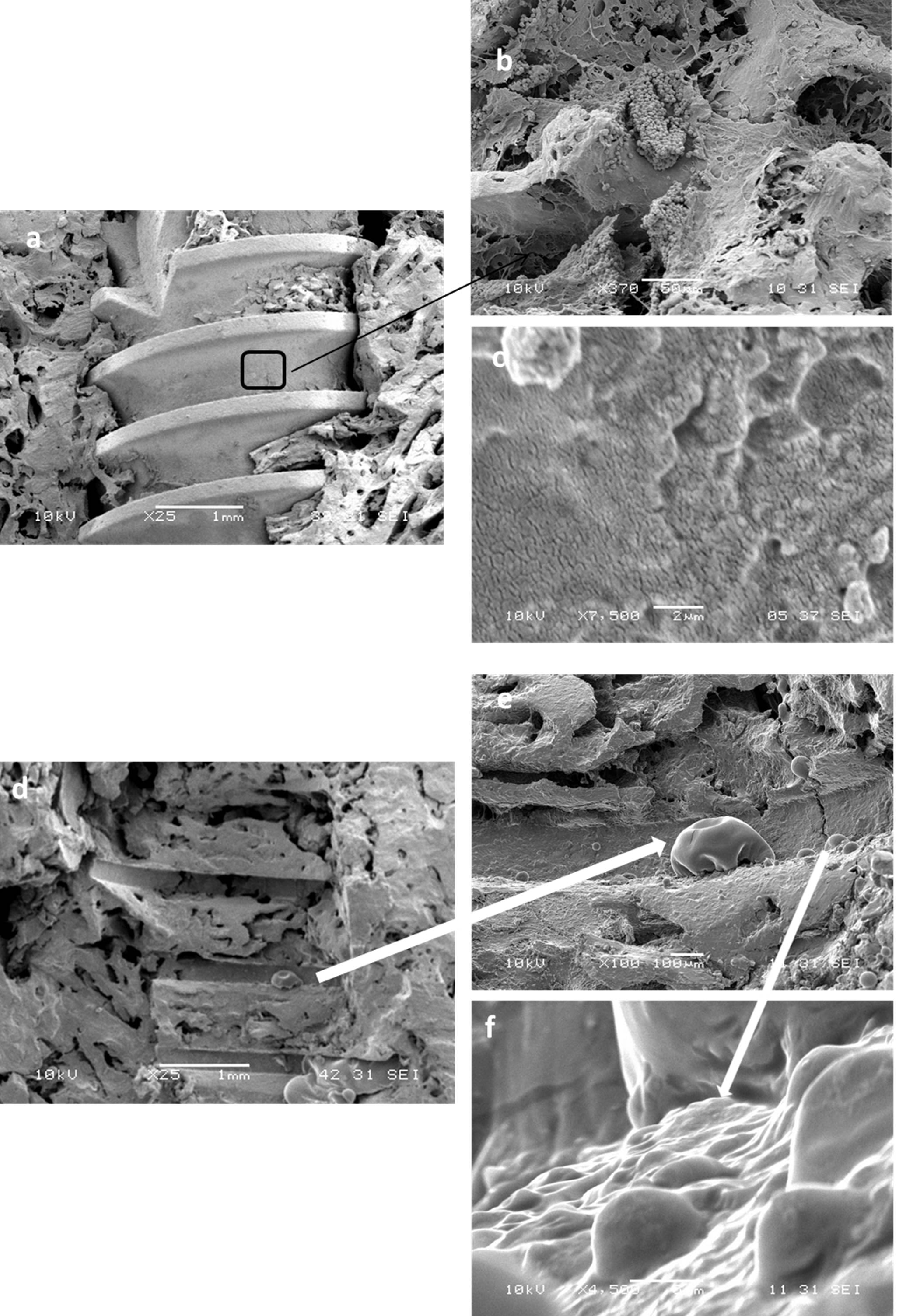


**Supplementary figure 3:**

Showing SEM imaging of scaffold/implant interface:

a, b & c: Surface of implant retrieved from socket grafted with TAMP-BG after two weeks:

a- Surface of implant serrations with the surrounding bone (SEM X25). b- Newly deposited bone matrix integrated at the implant surfaces showing intimate contact with degraded TAMP-BG (SEM X370).c- Bioactive surface of the degraded TAMP-BG particles covered with HCA (SEM X7500).d, e & f: The interface bone surface opposing the titanium fixture from socket grafted with TAMP-BG at two weeks intervals: d- The interface surface showing bone ridges within the remaining extraction socket area (SEM X25).e- Higher magnification of (d) indicating the presence of osteoclast like cells (white arrow) close to the osteoblasts (SEM X100).f- Higher magnification of the interface bone ridge indicating numerous osteogenic cells on bone matrix (white arrow) (SEM X4.500).

**Supplementary figure 4:**


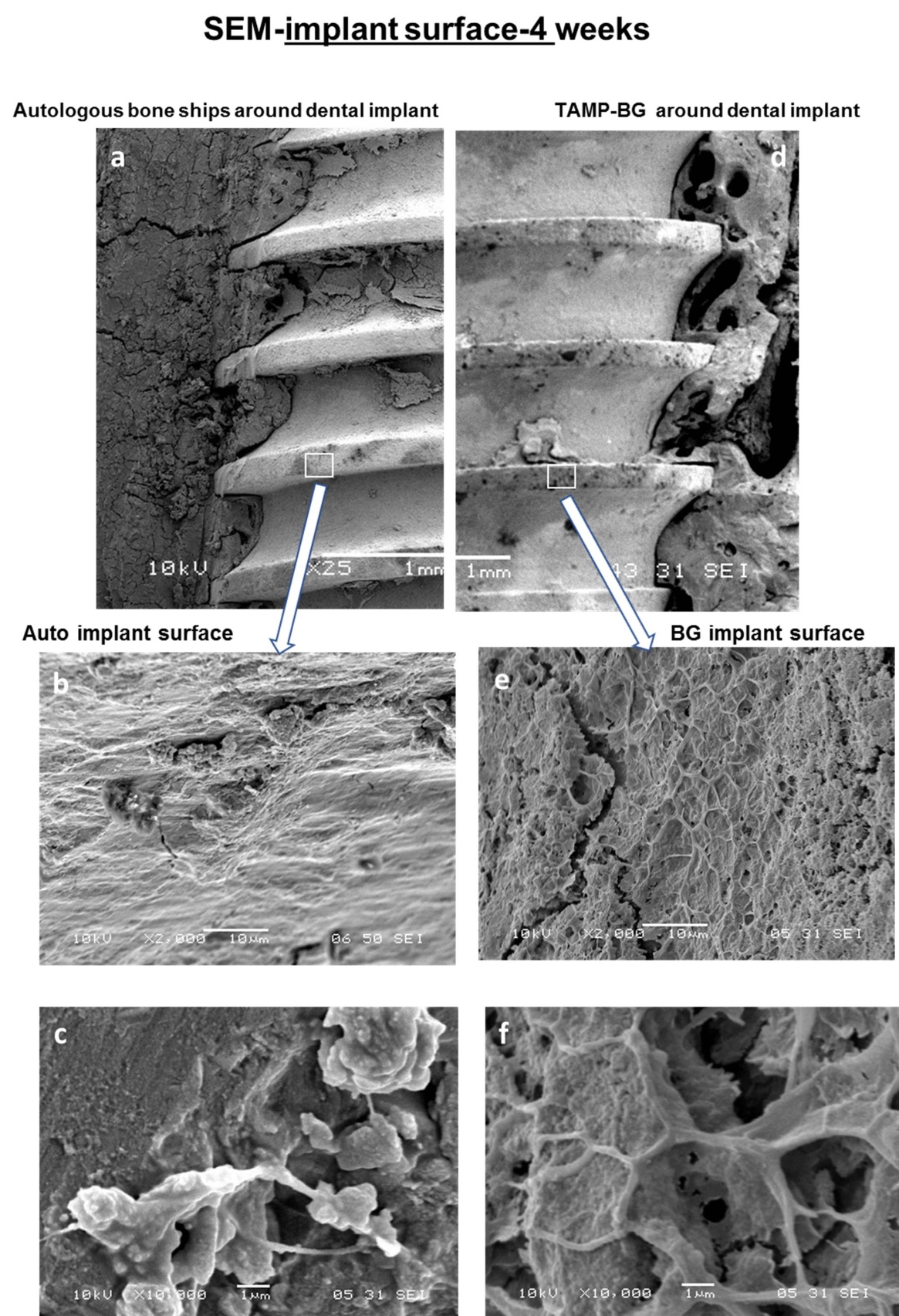


**Supplementary figure 4:**

Showing SEM imaging of bone formation pattern around dental implant at the interface of 4 weeks samples. a, b & c showing the interface of implant surface in sockets grafted with autologous bone chips.

a- Implant surface and osseointegrated bone (SEM X25).b- Implant surface at the serration representing the white square in (a) and indicating the solid bone deposition onto the implant surface (SEM X2000). c- Higher magnification of the bone matrix deposited onto the serration of the implant fixture (SEM X10, 000). d, e & f showing the interface of implant surface in sockets grafted with TAMP-BG particles. d- Implant surface and osseointegrated bone (SEM X25).e- Bone deposited onto the surface of the serration representing the white square in (d). It is following the same pattern of nano-macro porous bioglass scaffold (SEM X2000). f- Higher magnification of the bone matrix in (e) demonstrating the pattern of bone deposited onto the serration (SEM X10,000).

**Supplementary figure 5:**


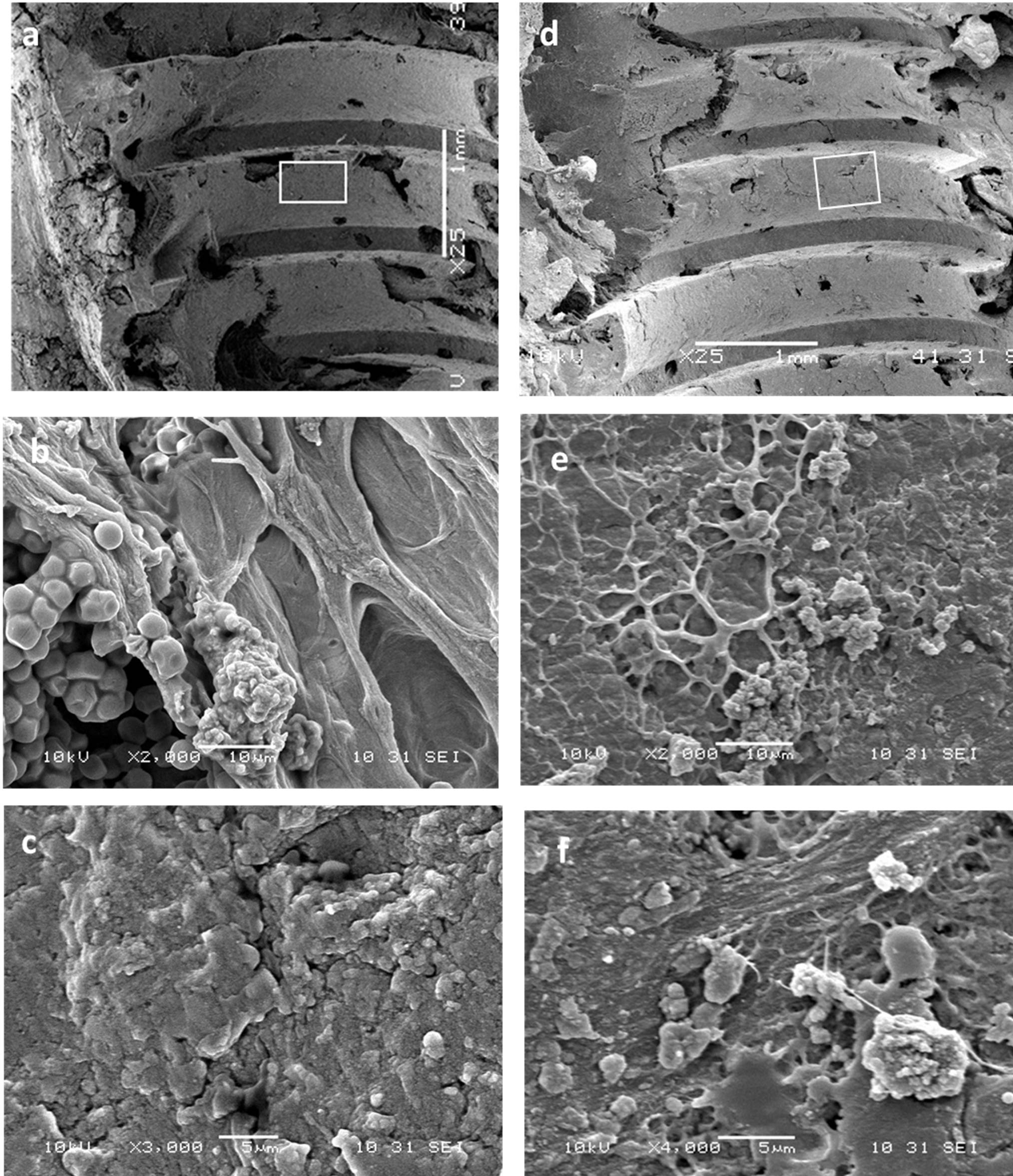


**Supplementary figure 5:**

Showing SEM imaging of bone surface interface opposing the titanium fixture showing the imprint of the implant on the bone surface at four weeks interval; a, b &c : The interface bone surface opposing titanium fixture in socket grafted with autologous bone chips

a- The interface showing bone ridges within the extraction socket area (SEM X25).b- Higher magnification of square area in (a) showing mineralized bone matrix as shown in crystals formation of hydroxyapatite (to the left)(SEM X2000). c- Larger magnification of newly formed bone that appears as a continuous layer representing the osseointegrated bone surface (SEM X 3000).d, e &f :The interface bone surface opposing titanium fixture in socket grafted with TAMP-BG particles. d-The interface showing bone ridges within the extraction socket area (SEM X25). e- Higher magnification of square area in (d) showing the pattern of new bone deposited on the socket walls following the same pattern of nano macro porous bioglass scaffold. Deposited mineralized nodules are seen aggregated (SEM X2000).f- Larger magnification of the same area seen in (e) showing the presence of osteogenic cells adjacent to the aggregated mineralized nodules (SEM X4000).

**Supplementary Fig. 6**

**
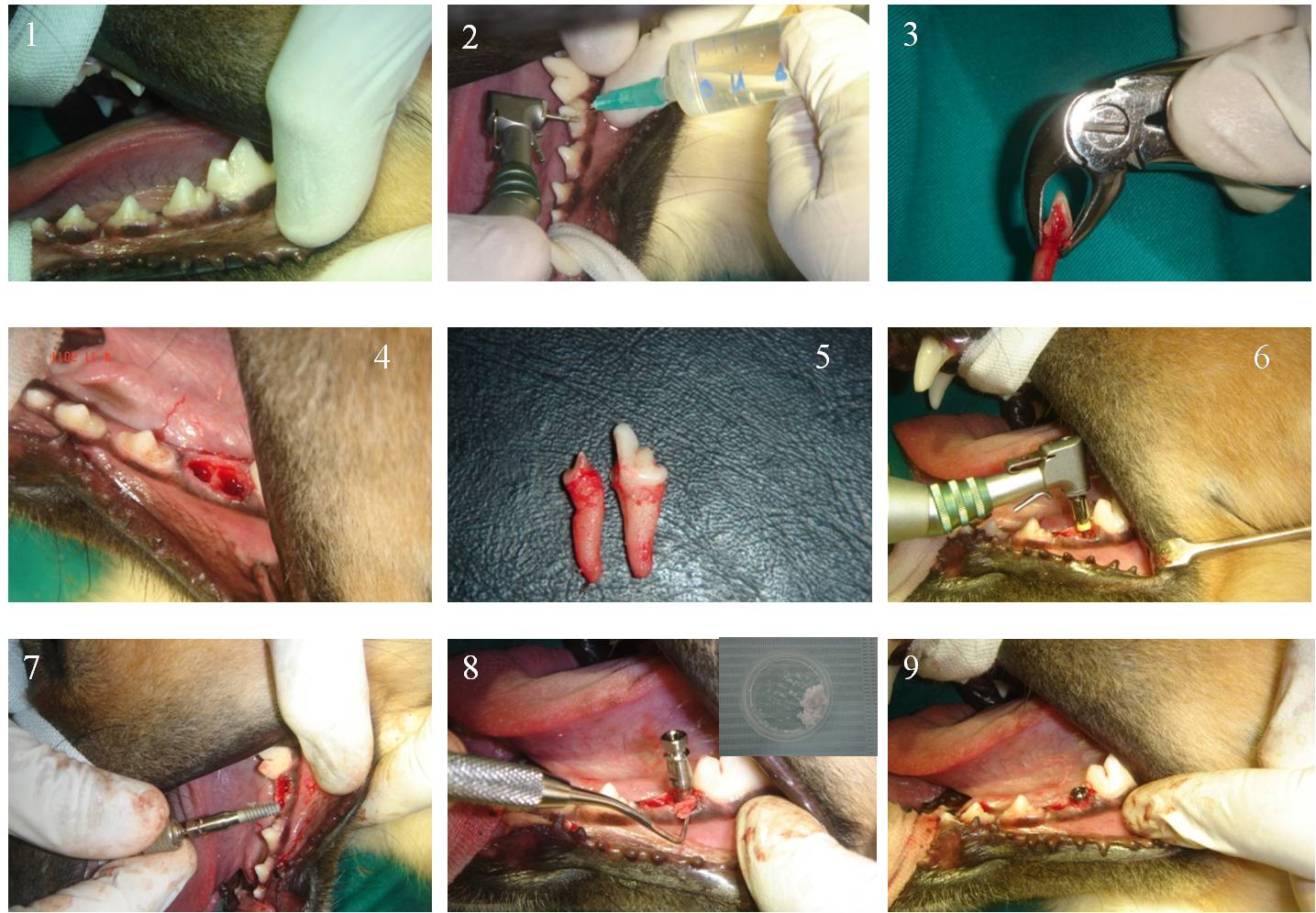
**

**Supplementary Fig. 6**

Showing trans-operative images; where mandibular fourth premolar (P4) in Dog was selected to be extracted (1).The tooth was planned to be surgically separated into two halves by diamond bur mounted on handpiece 13.000 rpm with external saline irrigation in order to be extracted atraumatically (2). The distal half of the crown along with the distal root was removed using English Style Forceps (3). Occlusal view of the socket showing intact gingival margin and interseptal bone denoting minimal surgical trauma (4). The mesial and distal roots were extracted intact (5). Implant osteotomy site was prepared under cooling system to receive a Titanium implant fixture of 8mm length and 3.7mm diameter (6). The implant was placed in the osteotomy site taking care not to be inserted to the full length (7). Bone graft particles were applied to the threads of the implants prior to be screwed to the full length with an insert of TAMP-BG particles mixed with saline (8). Post-operative view of the implant with bone graft particles filling the gap between the implant and the extraction socket wall (9).
